# *Candida auris*—“Ten Years After”

**DOI:** 10.3390/jof6010002

**Published:** 2019-12-18

**Authors:** Jacques F. Meis, Anuradha Chowdhary

**Affiliations:** 1 Department of Medical Microbiology and Infectious Diseases, Canisius-Wilhelmina Hospital, 6532 SZ Nijmegen, The Netherlands; 2 Centre of Expertise in Mycology Radboudumc, Canisius-Wilhelmina Hospital, 6532 SZ Nijmegen, The Netherlands; 3 Department of Medical Mycology, Vallabhbhai Patel Chest Institute, University of Delhi, Delhi 110007, India

We would like to thank all contributors to this Special Issue on *Candida auris*. We are extremely happy that we received eleven reviews/perspectives/original papers for publication.

This year marks the 10th anniversary of the first formal description of *Candida auris*, isolated from the external ear of a Japanese patient in 2009. In a relatively short period, *C. auris* was able to spread all over the world, mainly in hospitals, being reported currently from six continents and more than 40 countries. Investigators from the Centers for Disease Control and Prevention (CDC), Atlanta, USA, put forward a perspective as to how this pathogen may have been able to evolve [1] and proposed infection control measures to combat *C. auris* and clean hospital environments [2]. Several countries have reported persistent problems and prolonged outbreaks in healthcare facilities. A major outbreak in Oman [3] and experiences from the UK in handling *C. auris* outbreaks [4] are reported in this special issue. *C. auris* is easily transmitted in healthcare settings and is the first fungus to behave like a nosocomial bacterial pathogen with the potential to cause epidemics. It is, therefore, important to have patients nursed in contact isolation when they are potentially exposed to *C. auris* in distant healthcare institutions, as shown in examples from the Netherlands [5]. *C. auris* is the first fungal pathogen to be regarded as an urgent threat, side by side with carbapenem-resistant *Acinetobacter*, *Clostridioides difficile*, carbapenem-resistant Enterobacterales and drug-resistant *Neisseria gonorrhoeae* in the latest CDC report [6]. A major explanation for the rapid worldwide spread is that *C. auris* is often misidentified with phenotypic identification methods. To test laboratory preparedness, blinded *C. auris* strains were sent to clinical laboratories in the Netherlands, Belgium and Luxembourg. Disappointedly a significant portion of laboratories were unable to come up with a proper identification when using routine MALDI testing [7,8]. Molecular diagnostics may be the way to go especially, when enhanced surveillance for *C. auris* is needed to prevent transmission within and between hospitals and nursing home facilities [9]. More than 90% of *C. auris* isolates are fluconazole-resistant [10], with some rare isolates also being resistant to all three major antifungal classes, leaving no treatment options. Therefore, there is an urgent need to find new options for treating this pathogen and a new way is to look for activity of old drugs such as iodoquinol and miltefosine and reposition them as potential novel therapeutics for *C. auris* [11]. Much remains to be learned on the behaviour of *C. auris*. Diluca et al. [12] showed that *C. auris* has a completely different growth pattern compared to other *Candida* species by microcalorimetry. Finally, social media are potentially helpful in predicting emergence of *C. auris* in places before these findings are formally published [13]. All papers in this Special Issue have added to the increasing knowledge of *C. auris*, often with provocative hypotheses that will direct future research in respective field of study.

Again, we wish to thank all authors and reviewers for their significant contributions to this Special Issue and for making it a highly successful and timely collection of studies.
